# Takotsubo syndrome as an overlooked and elusive cause of a single episode of dyspnea in young women: a case report

**DOI:** 10.1186/s12872-021-02239-4

**Published:** 2021-09-10

**Authors:** Sung Eun Lee, Seung-Hyun Yoon, Hyo Jung Kang, Jung Hwan Ahn

**Affiliations:** 1 Department of Emergency Medicine, Ajou University School of Medicine, 164, World cup-ro, Yeongtong-gu, Suwon, 16499 Republic of Korea; 2 Department of Physical Medicine and Rehabilitation, Ajou University School of Medicine, Suwon, Republic of Korea; 3 Davos Hospital, Yongin, Republic of Korea

**Keywords:** Takotsubo syndrome, Dyspnea, Postoperative complications, Cardiac biomarker, Echocardiography

## Abstract

**Background:**

Dyspnea is a common symptom in patients presenting to the emergency department. It has a variety of causes that range from non-urgent to life-threatening. One episode of dyspnea in a healthy young person is easy to overlook. However, if the symptoms occur after physically or emotionally stressful events, careful evaluation needs to be undertaken because it may be associated with Takotsubo syndrome, which is rarely expected but can be fatal. Herein, we report the case of Takotsubo syndrome in a healthy young woman who arrived at the emergency department after experiencing a short single episode of dyspnea following a minor surgery.

**Case presentation:**

A 23-year old woman with no underlying chronic disease underwent closed reduction surgery for a nasal bone fracture under general anesthesia (with sevoflurane as the anesthetic). Approximately 5 h later, she presented to the emergency department with dyspnea, which improved soon upon arrival at the emergency department. There were no other symptoms. The dyspnea occurred about 5 h after being discharged on observation, with an uneventful postoperative course. Her electrocardiogram and chest X-ray findings were unremarkable. On testing, troponin I and creatine kinase myocardial band levels were elevated at 6.122 ng/mL and 11.2 µg/L (reference ranges: 0.000–0.046 ng/mL and 0.0–5.0 µg/L), respectively. Bedside echocardiography revealed an ejection fraction of 25%, with mid-ventricular and apical akinesia and basal hyperkinesia. The pulmonary and coronary angiographic computed tomographic scans were unremarkable. Hence, apical Takotsubo syndrome was suspected. A follow-up echocardiogram taken 5 days after admission showed full recovery with a normalized ejection fraction (60%) and no regional wall motion abnormality. The patient was discharged on the sixth day with no other complications.

**Conclusion:**

When atypical symptoms, such as transient dyspnea, manifest, it becomes necessary to suspect and diagnose Takotsubo syndrome to ensure timely and appropriate medical management, especially when a preceding stressful event, such as minor surgery has occurred. It might be helpful to perform bedside point-of-care echocardiography to check for regional wall motion abnormalities that are typically associated with Takotsubo syndrome.

**Supplementary Information:**

The online version contains supplementary material available at 10.1186/s12872-021-02239-4.

## Background

Dyspnea is a common symptom in patients presenting to the emergency department (ED) [1]. It has a variety of causes that range from non-urgent to life-threatening [1]. Unfortunately, the severity of dyspnea observed in the patient and the severity of the pathology behind the symptom do not correlate well [1]. Therefore, when a patient complains of dyspnea, physicians must always anticipate and prepare for all underlying pathologies of dyspnea that may soon develop into life-threatening complications. Risk factors including old age, immobilization, previous history of lung disease, acute coronary syndrome (ACS), and heart failure direct physicians to prepare for the worst-case scenarios. However, in a clinical setting, it is not usual to anticipate a rare and complex pathology such as Takotsubo syndrome (TS) in young, healthy patients with no risk factors [2–5]. Essentially, TS is characterized by acute cardiac dysfunction with a typical regional wall motion abnormality (RWMA), but unlike most other cardiac pathologies, patients achieve full recovery within a short period [2–4, 6, 7]. The clinical presentation can be varied, ranging from non-specific symptoms to a life-threatening presentation [4, 7–10]. Chest pain and dyspnea on exertion are the most common symptoms of TS, seen in 67–81% of patients, with chest pain being the prime symptom [2, 7, 11]. Almost 80–90% of patients with TS are elderly, postmenopausal women aged above 60 years [2, 4, 7, 11]. Therefore, diagnosing TS is challenging in healthy young patients who present with mild symptoms [2, 4, 7, 11]. Moreover, TS could be accompanied by life-threatening complications. Congestive heart failure (44–57% of cases), cardiogenic shock (15–45%), pulmonary edema, dysrhythmias (10%), thromboembolism, left ventricular wall rupture, and death have been reported as fatal complications of TS [10, 12, 13]. Hence, emergency care physicians should be aware of this unusual condition to ensure its timely, appropriate management.

We present a case of a previously healthy young woman who arrived at the ED after just one episode of dyspnea that lasted 30 min. The patient had received surgical treatment of a nasal bone fracture by closed reduction approximately 5 h prior to the onset of symptoms. Dyspnea improved on arrival at the ED. Further, no other symptoms were observed and no other complaints were noted in the ED. The symptoms alone made it difficult to suspect TS, but laboratory tests for safety disposition showed elevated cardiac enzymes. Further testing, including bedside echocardiogram, confirmed her TS. We report this rare case of TS because, to the best of our knowledge, there have been no other reports of TS in young women presenting with such mild, transient symptoms.

## Case presentation

**Fig. 1:**
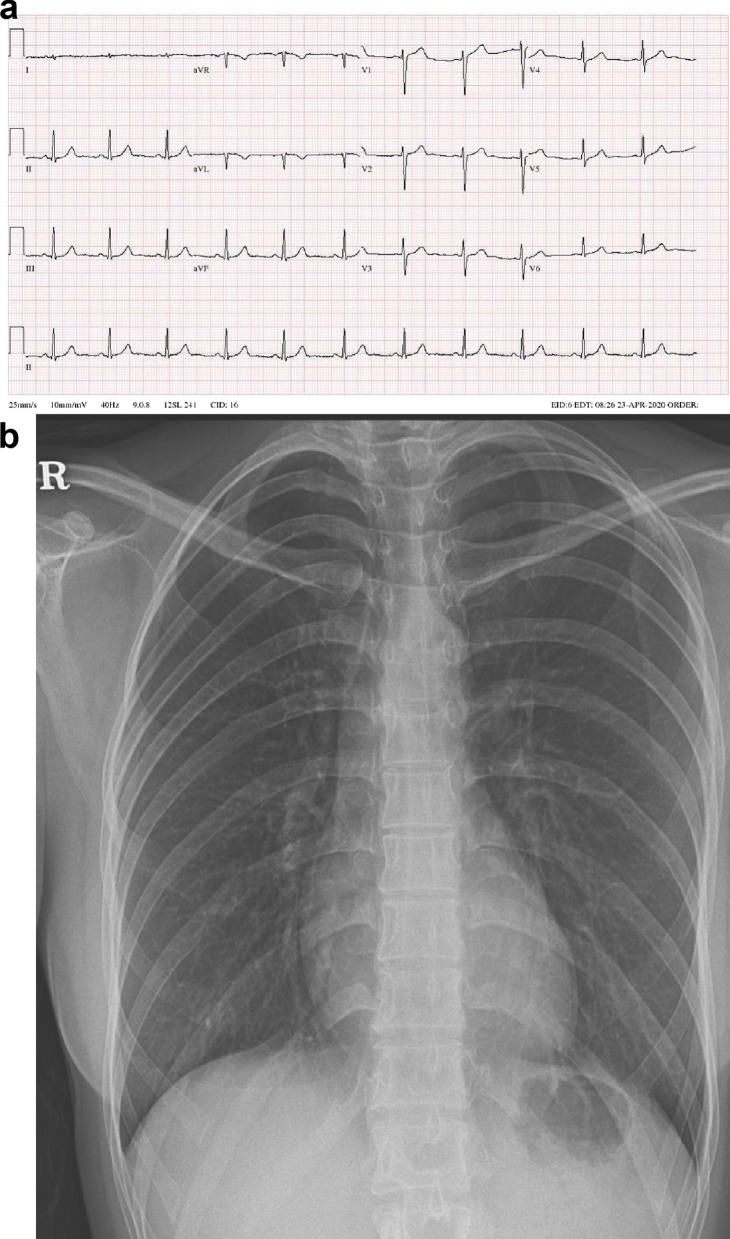
Electrocardiogram and chest radiography at admission in emergency department. **a** Electrocardiogram, showing regular sinus rhythm without any specific findings. **b** Chest radiography, showing no unusual findings

**Fig. 2:**
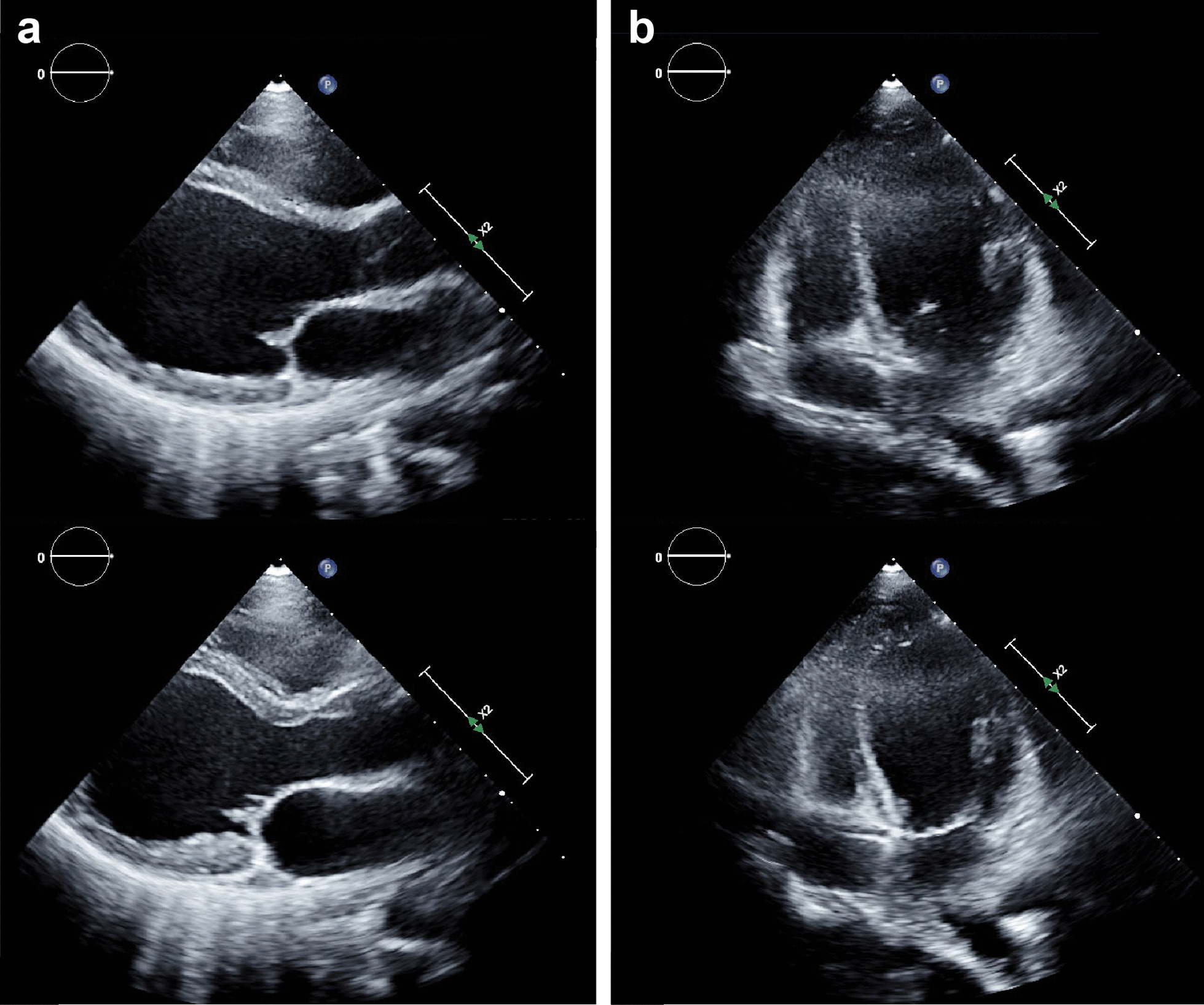
Initial transthoracic echocardiogram of end-diastolic and end-systolic phase. Mid-ventricular and apical akinesia with basal hyperkinesia are observed. **a** Parasternal long axis view of end-diastolic (upper) and end-systolic phase (lower). **b** Apical four chamber view of end-diastolic (upper) and end-systolic phase (lower)

**Fig. 3:**
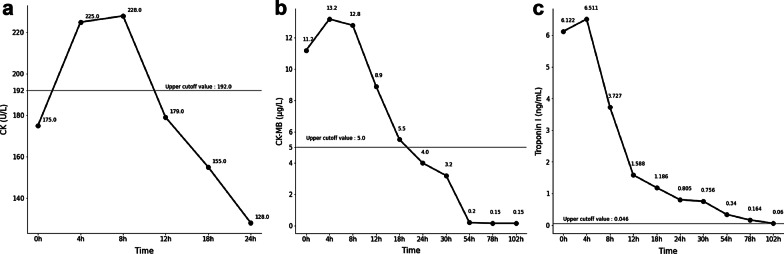
Time course of creatine kinase (**a**), creatine kinase myocardial band (**b**) and high-sensitive troponin I levels (**c**). Panels in this figure were made by matplotlib version 3.3.4 [14]. CK = creatine kinase, CK-MB = creatine kinase myocardial band

**Fig. 4:**
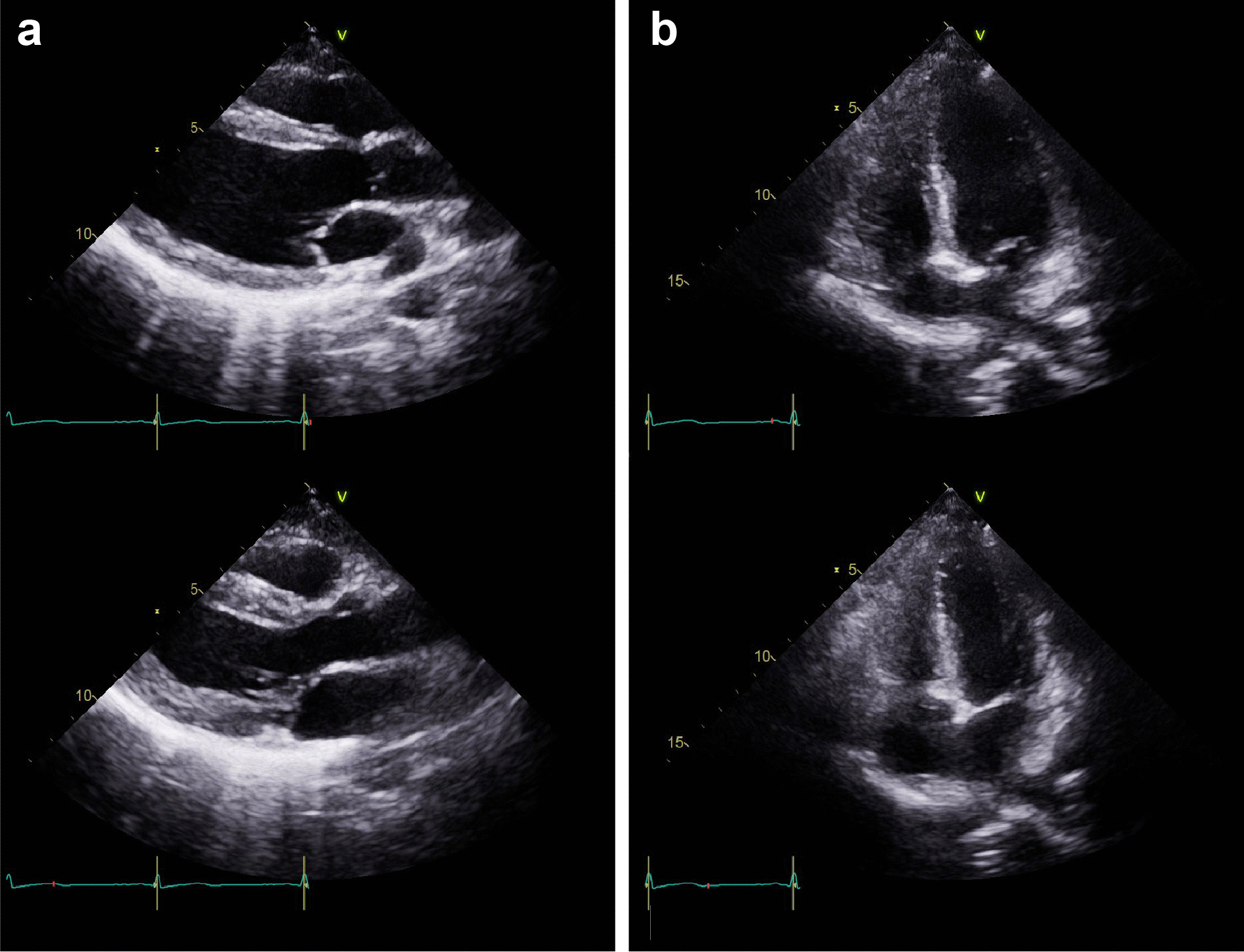
Follow up transthoracic echocardiogram at 5 days later of end-diastolic and end-systolic phase. Full recovery with normalized ejection fraction and no regional wall motion abnormality are observed. **a** Parasternal long axis view of end-diastolic (upper) and end-systolic phase (lower). **b** Apical four chamber view of end-diastolic (upper) and end-systolic phase (lower)

A 23-year-old woman with no underlying chronic disease presented to the ED after an episode of dyspnea. Her dyspnea improved upon arrival; she was, otherwise, symptom free. Her vitals upon arrival were stable: oxygen saturation, 100%; blood pressure, 121/83 mmHg; pulse rate, 105 beats/min; and respiratory rate, 16 breaths/min. Exactly 4 h and 40 min prior to the onset of symptoms, she had undergone a closed reduction surgery for a nasal bone fracture under general anesthesia. The course of surgery was uneventful and the patient was discharged after observation as she showed no unusual signs postoperatively. After arriving home, she was drinking a glass of water when the dyspnea started. This symptom lasted 30 min. She had no history of a recent upper respiratory infection or immobilization, did not smoke or drink alcohol, and had no family history of cardiac or cerebrovascular diseases. The physical examination showed no signs of neck vein engorgement or leg edema; there were also no unusual results pertaining to the lung, heart, or abdomen. Her electrocardiogram (ECG) and chest radiography did not show any unusual pathology (Fig. 1). Arterial blood gas analysis results were as follows: pH, 7.407; pCO_2_, 29.6 mmHg; pO_2_, 105.4 mmHg; base excess, − 4.1 mmol/L; HCO_3,_ 18.8 mmol/L; and O_2_ saturation, 98.2%. The laboratory investigations showed a normal blood cell count, and electrolyte and chemistry tests showed nothing abnormal except for a slightly elevated glucose level of 115 mg/dL (reference range: 74–106 mg/dL). From these findings, the initial diagnosis was that of an asthma attack or hyperventilation syndrome. Therefore, she was to be discharged and an appointment was fixed at the outpatient department of the pulmonology center. Thereafter, to rule out occult pneumothorax, pulmonary embolism, and the rare case of a coronary event in a young adult, we ordered for cardiac biomarker testing and performed a chest computed tomography (CT). Cardiac markers showed creatine kinase levels within the normal range at 175 U/L (reference range: 26–192 U/L), but creatine kinase myocardial band and high-sensitive troponin I levels were elevated at 11.2 µg/L (reference range: 0.0–5.0), and 6.122 ng/mL (reference range: 0.000–0.046), respectively. Subsequently, an echocardiogram and pulmonary and coronary angiography CT were ordered to consider or exclude myocarditis, pulmonary embolism, pericarditis, and coronary artery disease. Finally, the bedside echocardiogram performed at the ED showed an ejection fraction (EF) of 25% by the modified Simpson method and midventricular and apical akinesia with basal hyperkinesia, which were indicative of RWMA associated with TS (Fig. 2, Additional files 1 and 2: Video 1 and 2). The pulmonary and coronary angiographic CT scans showed no abnormal findings. Considering these results, apical TS was the most likely diagnosis. She was admitted to the intensive care unit. During her hospitalization, no symptoms of chest pain or dyspnea were presented, creatinine kinase myocardial band and high-sensitive troponin I level decreased gradually (Fig. 3), and a follow-up echocardiogram performed on the fifth day of hospitalization showed full recovery with a normalized EF of 60% and no RWMA (Fig. 4, Additional files 3 and 4: Video 3 and 4). The patient was discharged on the sixth day with no other complications.

## Discussion and conclusions

TS, also known as “stress-induced cardiomyopathy,” “apical ballooning syndrome,” or “broken heart syndrome” was reported in 1990 by Japanese cardiologists [6]. Because the systolic dysfunction of the left ventricle in affected patients is usually fully normalized, TS is considered a benign condition. However, recent studies and review articles reported that acute complications of TS may be comparable to those of ACS and myocarditis. Moreover, the incidence rate of shock and sudden death associated with TS are comparable to those from ACS and myocarditis [2, 10]. Therefore, physicians should be aware that such fatal complications can occur following TS. In a cohort of 136 patients with TS, 2.2% (3/136) of the patients died, 3.7% (5/136) developed apical thrombi, and 5.1% (7/136) developed shock [2]. Other review articles have reported mortality rates of 2%, thrombi formation rates of 2–5%, and cardiogenic shock rates of up to 10% [2, 4, 7, 10]. Hence, critical monitoring during the acute phase is necessary, and it is recommended that patients be admitted to intensive care unit until left ventricular function is normalized [4]. Unfortunately, as diagnosing TS is difficult, the incidence of TS is highly underestimated [2, 4, 7]. Therefore, it must be included in the differential diagnoses for patients with unexplained dyspnea, chest pain, hypotension, and/or elevated cardiac biomarker levels.

Various diagnostic criteria for TS have been developed. The most recent criteria that came into clinical practice after achieving consensus was announced by international TS investigators in 2018, and it is called the International Takotsubo Diagnostic Criteria (InterTAK Diagnostic Criteria) [15]. The InterTAK Diagnostic Criteria are as follows: RWMA that is appropriate for TS and returns to normal within a short period, existence of triggering factors, postmenopausal status (as this is the age predominantly affected), ECG changes, cardiac markers and brain natriuretic peptide level elevations, and exclusive differential diagnosis such as myocarditis [15].

When the symptoms of the patients fit the criteria, the diagnosis might be easy; however, atypical presentations such as normal electrocardiography or young age may challenge the confirmatory diagnosis of TS. Electrocardiograms of TS patients usually show similar findings that seen in myocardial infarction, such as ST segment elevations, ST segment depressions, or T wave inversions; however, they may also present with non-specific ECG changes, or even a normal ECG [2, 4]. In a study by Sharkey et al. [2], 17/136 cases (13%) showed a non-specific ECG and 2/136 cases (1%) showed normal ECGs. Troponin T level were varied, with a range of 0.01–5.2 ng/mL, but troponin T levels above 6.0 ng/mL or troponin I levels above 15 ng/mL were reported to be rare [11]. Cardiac brain natriuretic peptide levels can also be elevated [4, 7]. Although postmenopausal women (age: 60–70 years) are the most susceptible to TS, it has been diagnosed in young adults and men as well, albeit rarely; a case report of TS in a newborn has also been published [3–5, 12, 16–21]. TS in young patients is usually characterized by the occurrence of chest pain accompanied by ECG changes [16], severe arrhythmias [18, 19], and moderate-to-severe dyspnea requiring exogenous oxygen treatment [3], or the condition can present with non-specific symptoms with hemodynamic instability [12]. Different types of triggers exist for TS in the young age, including video games [16], participating in an angry debate [17], minor surgery with general anesthesia [12, 18], postpartum period [3], or having undergone inotropic treatment [19]. TS has been diagnosed especially among young women during the postpartum period [3, 20]. Minatoguchi et al. [3] reviewed 18 cases of TS, including 16 cases during the postpartum period and 2 cases of TS during pregnancy. In those reports, chest pain and dyspnea were the dominant symptoms, and most of those cases occurred within 24 h after delivery.

In our case, normal ECG findings, the young age of the woman, and only one episode of dyspnea challenged the diagnosis of TS despite the stressful event (surgery) and elevated levels of cardiac enzymes. Her arterial blood gas analysis showed hyperventilation but no signs of hypoxia, and her chest X-ray and ECG were normal. Therefore, our initial diagnoses were pneumothorax, asthma, or hyperventilation syndrome, and a cardiac cause was not considered, especially not a rare pathology such as TS. Cardiac biomarker testing was ordered to safely discharge the patient because, although rare, coronary events have been reported in young women with no risk factors. When the creatine kinase myocardial band and troponin I levels were found to be elevated, an echocardiography and CT of the coronary and pulmonary arteries were performed. However, even at this stage, we suspected the patient had a pulmonary embolism or acute myocardial infarction; TS was not suspected since the patient had no additional dyspnea or signs of EF reduced heart failure. Although cardiac magnetic resonance image with gadolinium enhancement was not implemented, the possibility of myocarditis was excluded for the following reasons: the patient had no symptom of infection within two weeks, no fever at the time of visit, and the inflammation markers were also within normal limits on laboratory tests. The findings for this patient were atypical considering our differential diagnosis, and therefore it was a difficult case to diagnose. According to the diagnostic flow of TS suggested by Napp and Bauersachs [4], in patients with no abnormally obstructed artery, TS may be diagnosed if the area of RWMA is similar to that of typical TS findings. If the findings are atypical, a magnetic resonance image must be performed. If there is no late gadolinium enhancement, the patient may be diagnosed with TS [4]. The apical RWMA finding typical of TS and the lack of abnormal CT findings led to the conclusion that the patient had developed TS. However, it is difficult to conclude that single episode of dyspnea is caused by TS. However, the authors strongly assume that TS is the most likely cause of trigger in the situations where there is a great lack of evidence of other differential diagnosis, as described above. The exact mechanism that causes the single episode of dyspnea by the TS is unknown. As the most likely mechanism, single episode of dyspnea is thought to have been caused by sudden cardiac dysfunction caused by TS, and subsequent disappearance of dyspnea is thought to have been caused by compensation mechanism. Therefore, it is assumed that the result of arterial blood gas analysis described in this case does not reflect arterial blood gas analysis pattern at the time of TS occurrence, and rather reflects the hyperventilation state after compensation.

To the best of our knowledge, till date, there have been no reports of a TS patient with a complaint of transient mild dyspnea without other symptoms, as in this case. In this case, TS was not suspected after observing only the patient’s symptom but was detected after observing the bedside echocardiogram results and noting the elevation of cardiac enzyme levels. In addition, since TS occurred after minor surgery, such as a closed reduction for a nasal bone fracture, this case shows that if a healthy young patient complains of dyspnea, detailed history taking is needed for detecting trigger factors.

When dyspnea occurs in young women, cardiovascular disease, including dilated cardiomyopathy and pulmonary hypertension are generally included in the differential diagnosis [22]. As in the present case, if dyspnea occurs after stressful events such as minor surgeries, TS should be included in the differential diagnosis as a cause of dyspnea. Moreover, TS should be carefully and timely diagnosed so that patients may receive proper management and care. However, regardless of the diagnostic criteria, diagnosing TS is difficult, especially in cases where it is not expected. What can be learned from this case is that when healthy young people with preceding stressful events present to the ED with a chief complaint of dyspnea, ECG should be performed and cardiac biomarkers should be tested considering TS as a differential diagnosis. It is not necessary to perform a full-spectrum echocardiography in all patients with a single episode of dyspnea, but if the dyspnea is accompanied by preceding stressful events, performing bedside point-of-care echocardiography while suspecting TS would further ensure patient safety, even if full-spectrum echocardiography is not performed. Further, it might be helpful to perform point-of-care echocardiography to check for RWMA that are typically associated with TS.

## Supplementary Information

**Additional file 1**. Initial echocardiography exam: parasternal long axis view showing mid-ventricular and apical akinesia with basal hyperkinesia.

**Additional file 2**. Initial echocardiography exam: apical four chamber view showing mid-ventricular and apical akinesia with basal hyperkinesia.

**Additional file 3**. Follow up echocardiography exam: parasternal long axis view showing full recovery with no regional wall motion abnormality.

**Additional file 4**. Follow up echocardiography exam: apical four chamber view showing full recovery with no regional wall motion abnormality.

## Data Availability

All data generated or analysed during this study are included in this published article (and its supplementary information files). The matplotlib library (version 3.3.4) to draw Fig. 3 used during the current study are available in the Zenodo repository, https://doi.org/10.5281/zenodo.4475376.

## Abbreviations

ED: Emergency department
ACS: Acute coronary syndrome
TS: Takotsubo syndrome
RWMA: Regional wall motion abnormality
ECG: Electrocardiogram
CT: Computed tomography
EF: Ejection fraction
